# Strengthening Marriages in Egypt: Impact of Divorce on Women

**DOI:** 10.3390/bs10010014

**Published:** 2019-12-25

**Authors:** Jaime E. Mendoza, Maram Tolba, Yasmine Saleh

**Affiliations:** Department of Psychology, American University in Cairo; AUC Ave, P.O. Box 74, New Cairo 11835, Egypt; maramt@aucegypt.edu (M.T.); yasminesaleh@aucegypt.edu (Y.S.)

**Keywords:** marriage, divorce, mental health, Egypt, Arab families, resilience, stigma, marital expectations, secret lives, counseling

## Abstract

Divorce rates have been increasing around the world, and the Middle East is not immune to this reality. This pilot study investigates the phenomenological experiences of divorce for 20 Egyptian females. The study is qualitative, using in-depth interviews asking 14 questions that address different aspects of struggling marriages and post-divorce life. Five main themes were derived using a conventional approach to content analysis. The themes are: Expectations before marriage, secret life, relational dynamics, mental health, and resilience. Several sub-themes were identified in each category. The themes and subthemes are discussed. What was surprising was that many women experienced a greater sense of resilience and level of empowerment as a result of their post-divorce process. Recommendations for future research are discussed, including a replication of the study with a more stratified sample group and inclusion of men prior to developing any interventions.

## 1. Introduction

Divorce rates have been on the rise in the United States and most European countries since the 1960s. As a result, there has been an influx of publications on the topic of divorce and marriage in the West [1]. The situation should be very different for the Middle East, especially in Egypt, given the collectivistic focus of the region. However, the rising divorce rate in Egypt has caused significant social disturbance, moving the government to try and implement programs to decrease the rate of couples divorcing. Whether economic conditions are contributing factors or the emotional stress resulting from arranged marriages, the fact of the matter remains the same: Divorce rates are increasing in Egypt. The rise in divorce rates has also been attributed to a change in the legal system that took effect in 2000 throughout Egypt (but mostly practiced in metropolis areas because of the difficulty enforcing new laws), which grants women a no-fault divorce “khula” [2]. Such law grants women the right to divorce by renouncing her right to any financial benefits she would normally receive. In 2005, Personal Status Law Coalition (PSL) was issued, extending the divorced mothers’ rights to child custody until their children (boys and girls) reach the age of 15 or if she remarries, in which case it goes to the grandparents [2]. 

The Egyptian Central Agency for Public Mobilization and Statistics (CAPMAS) reported a higher rate of divorce. Egypt has seen an 83% increase in divorce rates between 1996 and 2017. “The divorce rate stood at 1.2 per 1000 marriages in the period between 1996 and 1999, compared to a rate of 2.2 per 1000 marriages in 2015” [3]. In addition [3], about 200,000 married couples get a divorce every year, and research suggests that 40% of marriages end within the first five years. As of 2017, divorce rates in Egypt were reported to be between 39.3 and 60.7%, depending on rural and urban locations.

In spite of these staggering numbers, there are very few research studies analyzing the causes and systemic impact of divorce in the Middle East, and are almost nonexistent in Egypt. This research project aimed to understand the phenomenon of divorce in Egypt, to find answers that are culturally (Arab) and contextually (Middle East) appropriate to address the ultimate goal of developing guidelines to strengthen Arab marriages.

### 1.1. Causes of Divorce

Understanding the underlying reasons for divorce is instrumental to direct intervention efforts. One of the most cited reasons for divorce in the Arab world and other countries is early age of marriage [4,5]. Marrying at an early age is encouraged by Arab culture, but it is also a major risk factor for marital dissolution because the individuals are (1) not mature enough, (2) may not have full decision making, (3) are less financially stable, and (4) may have had minimal educational progress [6]. Al-Saadani [6] cites that 50% of women in the Arab region were first married before the age of 20. This was the case in many Arab countries, including Egypt, Sudan, Jordan, and Lebanon. In 2010, 39% of women who divorced in the UAE were between the ages of 20–25 [4]. In Egypt, most divorces among men happen between the ages of 30–35, and among women, the probability of getting a divorce is highest between the ages of 25–30 [7].

Educational attainment has been cited as a risk factor that influences marital stability. The higher the education, the more stable the marriage is [5,6,7]. Research shows that individuals with higher education show better judgement when selecting a spouse, are better at communication and managing conflict, as well as have more control over external interference particularly from family [5,7]. Moreover, in Jordanian studies found that among the main causes for divorce are lack of communication, incompatibility, lack of education, and a short courtship before marriage (Barhoum; Al Qaisi and Al-Majali; Al-Ghazwi as cited in [4]).

Additionally, family interference has often been cited as a factor that threatens the stability of the marriage. The family often has a say about the choice of spouse, up to interfering in the couple’s daily affairs [4,8,9]. Furthermore, over-involvement of the family, particularly the mother-in-law, contributes greatly to the dissolution of the marriage [4]. In a Palestinian study, 200 cases of divorce were brought to court because of an over-involved mother-in-law [10].

### 1.2. Expectations About Marriage

Among the factors influencing the rising divorce rates in the Middle East is the change brought about by urbanization [11,12]. This shift has led to a change in expectations of marriage from the more traditional family values and gender roles [12]. Today, “incompatibility” is reason enough for divorce. This was not the case in the past, where divorce was seen as a failure, irresponsibility, and a deviation from cultural norms [12]. Other changes are a shift in the gender role expectations of men and women, including increased educational attainment for women [11]. Although higher educational attainment is often correlated with more stable marriages, in the Middle East, higher education and increased employment for women has caused a change in their role in society [13]. Women can now leave marriages and support their children without having to move back into their parents’ homes because of financial needs [12]. Moreover, the changes in expectations of marriage include factors like how the couple spends their leisure time, division of labor, interference of family, and career choice [8]. These differences in expectations create a gap between the couple threatening the stability of the marriage. The changes brought about by urbanization and the new status for women rock the stability of the marriage.

### 1.3. Impact of Divorce on Mental Health

The stress of divorce influences individuals’ mental health. Despite the fact that divorce usually ends a dysfunctional relationship, this is often accompanied with feelings of helplessness, aggression, sadness, guilt, loneliness, and uncertainty about the possibility of resolution of the marriage (Angelisti, as cited in [13]). What makes the psychological stress of divorce even worse is the social stigma faced, mostly by women. In some Arab countries, divorce has been cited as “the worst trauma” a woman can endure [4]. Arab women describe the social stigma of being divorced as feeling criminalized, isolated from society, being blamed, being made to feel sinful, and having unequal rights [14]. In addition, divorced women are often seen as bad parents and bad spouses more so than men [8]. This leads many women to feel a great deal of regret [14]. Divorced women also tend to feel more emotionally empty, suffer from self-esteem issues, and may grow to be more depressed [14]. The study by Cohen and Savaya [8] showed that the more an individual feels stigmatized by society, the more difficult it is for them to adjust to post-divorce life.

### 1.4. Stigma

Although social changes in society are making divorce a more acceptable solution to marital problems, divorced individuals, even in the West, are often seen as “of a less desired kind ... reduced in our minds from a whole and usual person to a tainted, discounted one” [15]. Gerstel [15] emphasizes that the contextual factors of divorce, rather than divorce itself, are the ones that lead to disapproval and social stigmatization. The author highlights that women with children were criticized more often for divorce than women without children. Moreover, one of the issues divorced individuals deal with in the aftermath of divorce is the change in their social setting. Often, this means loss of friends that were common among the couple [12,15]. Such splitting of friends leads divorced individuals to be impelled to explain, blame, or defend their reasons for divorce [15]. This splitting is taken further when married friends avoid or reject the divorced individual; usually because the divorce threatens the still married couple, leaving the divorced individual feeling even more isolated and rejected [15].

Similarly, in the Arab world, despite the increased rate of divorce, it continues to be stigmatized [15]. Arab women are often seen as though they are broken, and yet are ostracized out of fear of their seduction [8,16]. These social ramifications often leave women enduring dysfunctional and abusive marriages in order to avoid this social discrimination and humiliation [8,16]. In a study that explores the challenges of divorce among Saudi women, women describe feeling excluded and not belonging to society, being blamed for the failure of their marriage, being seen as sinful and criminal-like, and dealing with inequity in a male-dominated society [14]. Due to the social stigma women face, some prefer to avoid revealing their marital status [14]. Divorce has been described as being “traumatic” for women due to financial challenges and decreased social status [16]. Arab women are also often seen as threatening the stability of other marriages, and are sexually objectified [15,16]. Moreover, women often experience conflict with their families post-divorce [14,16]. This conflict comes in the form of familial imposed restrictions and control, as well as the continued blame for the dissolution of the marriage [14,16]. Such social consequences faced by women hinder their post-divorce adjustment (Amato, as cited in [8]).

### 1.5. Resilience Post-Divorce

The consequences of divorce are often detrimental and long-lasting. Divorced individuals suffer from an array of physical and psychological distress [17]. As mentioned above, some Middle Eastern women experience divorce as “traumatic” [4], thus much of the research covers the negative consequences of divorce. However, there are some resilience factors to be considered in the face of divorce [17]. Research has shown that when faced with trauma, most people experience some positive consequences in the form of posttraumatic growth (Christopher as cited in [18]). In their research, Krumrei et al. [18] found that more people experience this growth when they utilize spiritual coping mechanisms. Moreover, research has shown that effectiveness in managing post-divorce conflict influences the degree of stressors following the dissolution. In many cases, divorce is seen as a way to leave a situation that was already painful, and despite experiencing grief, individuals also experience freedom, happiness, and a reinvention of the self (Tashiro and Frazier as cited in [17]). In addition, resilience amidst divorce is also a result of previous relationship quality, the support one experiences, the person’s worldview, and contextual factors (Bonanno, Wortman, and Nesse as cited in [17]). Finally, the more empathic the divorce process is, the more it protects the individuals’ identity and the more resilient they are able to be in the face of dissolution [17].

### 1.6. Limited Research, Particularly from the Middle East

Most research concerning divorce and separation comes from the United States. A review of articles from January 2000 to February 2016, shows that 22,360 research articles appeared dealing with the impact of separation and divorce, with very few addressing the repercussions and intervention approaches. During this same 16-year period, the number of articles focusing on divorce and separation in Arab countries was significantly lower at 1452. The number of published articles is even smaller, under 900 (897), when we focus on Egypt. The number of articles decreases significantly (76) when we look at resilience after divorce or separation.

## 2. Methodology

This qualitative study is grounded in 20 semi-structured in-depth interviews. A qualitative design requires a significantly lower number of participants because the goal is to achieve saturation of the phenomena of marriage and divorce in Egyptian women. This is consistent with the research cited in Guetterman [19], which states that in order to reach saturation, a minimum of 10 and a median of 28 is needed in qualitative research.

The participants were recruited through social networks and one social media posting. The interview comprised 14 questions based on a literature review and questions that were not addressed in other studies. The questions are divided into three sections: (1) Demographic information such as: Years of marriage, years since divorce, and number of children; (2) themes such as expectations before marriage, reasons for divorce, mental health, social stigma, the legal process of divorce; and (3) the positive consequences of divorce. Some interview questions have imbedded or clarifying questions, in case the participant did not provide a full answer.

The study was approved by the university’s Institutional Review Board (case #2018-2019-053) prior to any data collection. The participants were recruited through social networks and one social media posting. Each interview was conducted in either Arabic or English, depending on the participant’s preferred language. All participants signed an informed consent detailing the scope of the project and publications. All interviews were audio recorded and transcribed by teaching assistants. If the interview was in Arabic, it was translated during the transcription process. Once the transcripts were ready, we used a qualitative conventional approach content analysis and theme analysis of the interviews, consistent with Morse and Field [20] and Graneheim and Lundman’s [21] approach. We categorized and coded verbal data, classified it into multiple themes, and tabulated the information.

## 3. Results

The study took place from January 2019 through March 2019. The 20 participants were females with an age range of 26–52. All of the participants were from an upper–middle social class and most lived in Cairo, with only two participants from outside of Cairo. The number of years of marriage ranged from 1 year to 20+ years. The content analysis revealed five general themes: (1) Expectations before marriage, (2) secret life, (3) relational dynamics, (4) mental health, and (5) resilience. Each theme and sub-theme is depicted in Table 1 to provide greater clarity for each category.

### 3.1. Expectations before Marriage

One of the interview questions sought to understand the participants’ expectations before marriage. Participants discussed what they thought marriage would look like. Participants had a romantic and idealistic view of marriage. Subthemes in this category include romance, responsibility, appreciation, individuation, and division of roles. Before marriage, participants expected that there would be shared responsibility between husband and wife. This sharing informs the intimacy that is to be created between the two, as well as the gender role expectations each partner has of themselves and the other. One participant stated: “I was young and naive. I was optimistic. I thought marriage would be full of love and full of happiness. People sharing with each other, loving each other, being kind to each other. Helping each other. I thought the more you gave to your partner the more he appreciated you.”

### 3.2. Secret Life

Among the most common themes present in the interviews was men having secret lives outside of marriage. This was experienced in different forms, such as: A second relationship, drug addiction, porn addiction, and domestic violence. This secret life usually played a major factor in slowly destroying the marriage, and ultimately in providing the tipping point that drove the women in our sample to seek divorce. For example, in a marriage where violence and drug addiction were prevalent, the person stated: “He did not disclose his drug addiction nor his medical condition until much later in the marriage”. Furthermore, in many of the stories, women struggled with the discovery of a secret relationship from the husband’s side. One participant relates: “I thought I would go on with my life and try to forget it and try to let things pass and endure and so on. And I thought maybe he would leave her after a few months or so”.

### 3.3. Relational Dynamics

Understanding the relationship dynamics when the participants were married resulted in several sub-themes emerging. These include: Disengagement early in the relationship, under-involvement (by the male), minimal reciprocal communication, issues of power and control, and disappointment after marriage. A common identified pattern in the interviews is detachment early on in the relationship. This disentanglement could either be emotional, physical, or both, and can happen as early as during the time of engagement. One participant described this detachment as: “He didn’t kiss because I didn’t enjoy it. So we stopped kissing. I never enjoyed it. And hugging, I never felt safe there. He was never there. Even from the beginning.” Such disengagement leads to minimal reciprocal communication, where either one partner tries to seek the other, or both lead parallel lives with very little interaction.

Furthermore, one of the most common patterns was the under-involvement of the male, both as a husband and father. This dynamic often left the women feeling alone, which was one of the factors that paved the way for their decision to divorce. One participant stated “I did not feel I have a man in my life. It was a huge burden.”

Power and control were also among the common relational dynamics identified in the interviews. In an attempt to gain control and power in the relationship, women would either abstain from sexual relations or involve a powerful family member. On the other hand, men would either become physically abusive or abstain from providing financial support. All of these dynamics contributed to great disappointment after marriage and led the women to feel more hopeless about the future of the relationship.

### 3.4. Mental Health

Because our research seeks to understand post-divorce life, one of the aspects we focused on was mental health. Several sub-themes emerged, including: Depression, anger, worthlessness, helplessness, anxiety, and pursuit of psychological help in an attempt to resolve the marital disconnection. The majority of participants expressed feeling depressed, anxious, or both at some point during their marriage. Other emotions such as anger and worthlessness were reported during marriage and after divorce, particularly if there was an extramarital affair. Such emotions peaked and became quite intense just as the marriage was ending and during the immediate aftermath of divorce. One participant stated: “I went to a psychiatrist and was on antidepressants. It was a very hard phase for me and it hurts as if I was building bricks and then I destroyed all this effort and threw it in the garbage.” However, most of the participants usually reached a more normalized emotional state 9–18 months after divorce. Many of the participants sought mental health treatment either during the marriage or post-divorce. What was identified in the interviews was that many of the treatment recommendations by therapists or coaches during the marriage were often not therapeutically accurate or were culturally biased against the women. Statements like “endure the relationship” and comments that prescribe cultural gender expectations such as “women are supposed to make their husband happy, so you may not be doing your job correctly” were used. Thus, the positive impact of help-seeking on the relationship was minimal or nonexistent.

### 3.5. Resilience

The last question in our interview addresses the positive consequences of the divorce. The most frequent answer to this question was “freedom.” Participants often gained a sense of freedom, which allowed them to return to their forgotten self-identity and allowed for personal growth. Many responses included statements like: “I became me again;” “Freedom, I can be a human;” “I became stronger, I discovered things about myself”. Among the common responses that existed, especially with individuals who had experienced domestic violence or extramarital affairs in their marriages, were inner peace and a recovered sense of safety. Many women expressed that they felt empowered after going through the divorce and independently caring for themselves and their children emotionally and financially. Through freedom, a recuperated sense of self, safety, and empowerment, many participants experienced a sense of renewed hope for the future, both for themselves as individuals and for possible future relationships.

## 4. Discussion and Conclusions

The rising divorce rates in Egypt makes divorce a modern-day reality and phenomenon that we can no longer ignore or simply import Western interventions to support Arab families. In this study, we attempted to understand the lived experiences of divorced Egyptian women, to learn about specific areas of their marriages and their post-divorce life. The five themes that emerged in this study are useful in guiding further research. Although questions about stigma were asked, most of the participants did not experience long-term impact from getting divorced. In fact, many of the females reported that their struggle post-divorce lasted between 9 to 18 months. Afterwards, they were very open to sharing their marital status. As a result, stigma became a subtheme instead of a core theme that they experienced. What was surprising was that many women developed a greater sense of resilience as a result of their post-divorce process.

*Implications:*

The next step for this project is to replicate this study with a more diverse population sample. It is imperative that such research includes men and other participants from different socioeconomic groups, specifically a stratified sample, doing so will help us establish clarity on how men and women are impacted by divorce and socioeconomic status. This information will afford an opportunity in developing psychoeducational material that is culturally sensitive and culturally derived, so that future interventions may be created. These interventions can help support the growing number of individuals who are navigating post-divorce life. The material will also inform and educate those who are currently struggling in their marriage. Our findings provide significant guiding principles for mental health professionals as they treat couples. As discovered in the emerging themes, mental health professionals in Egypt are often ill-equipped to deal with struggling married couples or those experiencing challenges adjusting to a life after divorce. Our research can help provide important data that can be used in training professionals in how to help those struggling in their marriage. Such data lay the foundation of what happens in Egyptian marriages and what can be done about it.

*Limitations:*

Among the limitations of our research is that the sample size is not representative of the population of greater Cairo. Moreover, because our sample was all women, our findings only relate to the experiences of women. Having equal representation of men and women is essential in future research. In addition, our sample is all from an upper–middle social class. This may not be an accurate representation of the Egyptian population. Thus, for future expansion of this project, it is important to include a stratified sample for a more accurate presentation of Egyptian culture.

## Figures and Tables

**Table 1:** Emerging themes and subthemes.

Number	Theme	Subthemes
1	Expectations before marriage	Romance, responsibility, appreciation, individuation, and roles.
2	Secret life	Second relationship, drug addiction, porn addiction, and domestic violence.
3	Relational dynamics	Disengagement early in relationship, under-involvement (by male), minimal reciprocal communication, power and control, aggression, and disappointment after marriage. Prior to marriage communication: Moderate-to-high interaction. During marriage, moderate-to-minimal interaction.
4	Mental health	Depression, anger, worthlessness, helplessness, anxiety, and seeking psychological treatment.
5	Resilience	Freedom, personal development, re-capturing self-identity, inner peace, single parenting, safety, empowerment, self-care, and hope.
